# A practical implementation science framework for education

**DOI:** 10.1038/s41539-026-00430-w

**Published:** 2026-06-18

**Authors:** Scott E. Allen, Lisa Bardach, Jamie Jirout, Allyson Mackey, Dana McCoy, Luca Maria Pesando, René F. Kizilcec

**Affiliations:** 1 Department of Physics, Cornell University, Ithaca, NY USA; 2 Department of Psychology, Justus-Liebig University, Giessen, Germany; 3 School of Education and Human Development, University of Virginia, Charlottesville, VA USA; 4 Department of Psychology, University of Pennsylvania, Philadelphia, PA USA; 5 Human Development and Urban Education Advancement, Harvard University, Cambridge, MA USA; 6 Department of Social Research and Public Policy, New York University, Abu Dhabi, UAE; 7 Department of Information Science, Cornell University, Ithaca, NY USA

**Keywords:** Education, Learning and memory

## Abstract

Implementing new technologies and practices in schools is complex, but these efforts often fail in predictable ways. Implementation Science offers clear criteria for conducting a well-planned project and identifying the “known unknowns” to proactively anticipate likely complications, but its frameworks are complex and difficult to use. We present a simplified, user-friendly protocol for implementing evidence-based pedagogical practices and technologies in real-world educational settings.

## From promising application to successful implementation

A few years before his death, the physicist Heinrich Hertz confidently stated, “I do not think that the wireless [electromagnetic] waves I have discovered will have any practical application…It’s of no use whatsoever”^1^. In any scientific domain, translating basic science into applied science is not made difficult *only* by regulatory hurdles, professional pressures, funding processes, and editorial standards, among other factors. Even when the basic science is definitive, it is not immediately obvious how to apply it in the practical space. While it took only 10 years for applied radio technology to arise from Hertz’s discovery of electromagnetic waves, the same pace of progress is not usually achieved in most scientific domains, including the science of learning. Though “active learning” is often thought of as a feature of modern pedagogy, key components of the underlying basic science were discovered nearly 100 years ago. The *testing effect*, where learners take frequent formative quizzes, was observed as early as the 1910’s^2,3^, but was not widely utilized in applied educational research until the 1960’s^4^, and widespread adoption did not begin until the 1990’s^5^. In many educational settings, this and other centuries-old scientific contributions are still being gradually implemented to benefit students’ learning^6,7^. Too often, the enormous scientific and practical value of translating basic research into applied research is unrealized due to equally large scientific and practical challenges^8,9^.

**Fig. 1:**
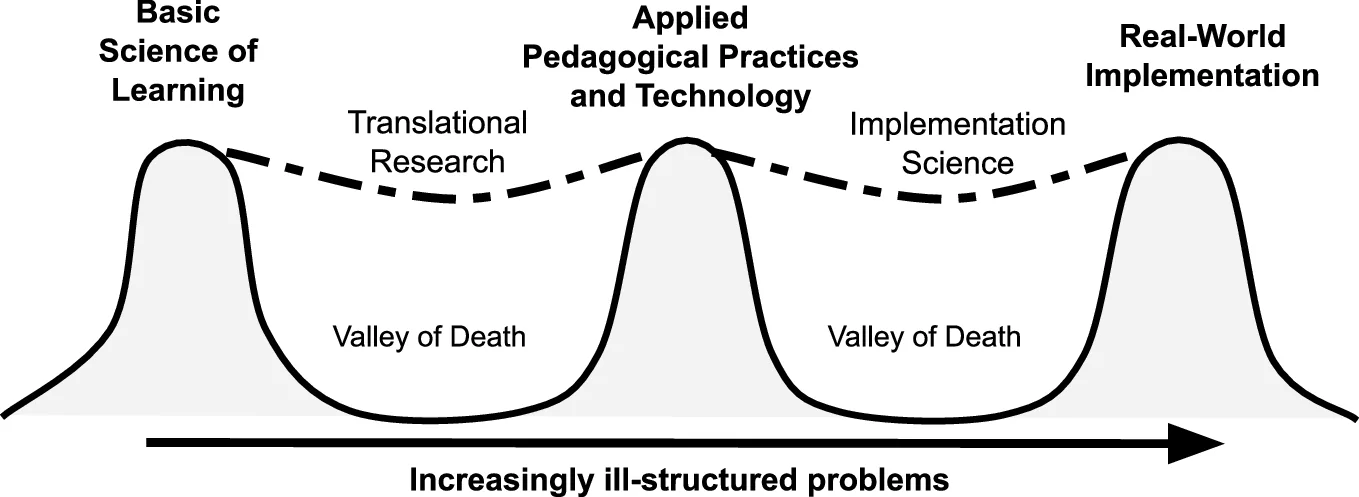
The separation between research domains is often depicted as the “Valley of Death”. The work of advancing the basic science of human learning, developing applied pedagogical practices and technologies, and implementing improvements in schools is usually conducted by completely isolated professional communities with separate funding sources and publication venues. In addition to the regulatory hurdles and professional pressures that prevent crossing the valleys between these three important scientific pursuits, the task of doing so is a legitimately challenging intellectual and practical struggle. The struggle is magnified by the increasingly ill-structured nature of the problems to solve. These problems tend to involve more numerous stakeholders, goal ambiguity, constraint volatility, and value conflict. Methodological tools are available to address these challenges. Translational Research provides the tools for the first valley. Implementation Science provides the tools for the second. Using these tools effectively is best achieved by attending to the ill-structured problems that arise in any effort to cross these valleys.

The treacherous gap between basic science and applied science is only the first half of the problem. Once an effective applied solution (i.e., practices, strategies, technologies, tools) is developed, its impact is not truly realized until it is implemented into socio-cultural systems and organizations^10^. Many implementation efforts are poorly planned, fail to adapt to typical challenges, and alienate local students and teachers when they should form partnerships with them^5,6,11,12^. This second gap from application to implementation is no less treacherous than the first. Each of these gaps in translation has been characterized as a “Valley of Death,” where profound scientific contributions go to die without yielding any real-world impact (Fig. 1)^13–15^. The *ill-structured* problems that arise in trying to cross these valleys should not be underestimated or ignored. Ill-structured problems entail conflicting definitions of success, diverse stakeholder interests, complex causal relationships, ambiguous data, and intricate human factors^16^. Any effort to conduct a real-world implementation should be ready to account for these challenging aspects of ill-structured problems. Approaching this important work of real-world implementation as though it will only present well-structured problems is a recipe for failure.

It would be a mistake to assume that once a practice is vetted by applied research, the translation to organizational implementation is merely a matter of scaling. Even when these applied solutions work as expected and are implemented in the same context in which they were originally developed and tested, assimilating them into existing educational organizations is notoriously difficult^17^. Moreover, even motivated and well-funded projects tend to fall into the same consistent failure modes^18,19^. Based on our synthesis of prior research^10,20,21^, we identify simplistic planning, lack of adaptation, and poor teamwork to be the most common reasons that implementation projects fail. Importantly, these are the same failure modes that tend to arise when solving any ill-structured problem^22,23^. Thus, we are optimistic that within this pattern of failure, there is an opportunity. To break out of these failure modes, the education research community needs (1) clear criteria for judging strong plans, (2) a set of known unknowns for anticipating challenges, and (3) user-friendly protocols for conducting implementation projects (see Supplementary Information). Fortunately, we do not need to develop these from scratch. An entire research discipline has emerged to meet this challenge of bridging the second gap, aptly named Implementation Science. Although initially developed in the healthcare context, Implementation Science has since been applied to many other social contexts, including education. In this piece, we will describe how findings from Implementation Science provide insights into why and how implementation efforts tend to fail, and offer consistent principles for improving rates of success. We will argue that the clear criteria and known unknowns from Implementation Science are mature enough to be used for planning and conducting implementation projects and that there is value in framing implementation projects as ill-structured problems. We provide a simplified protocol derived from Implementation Science to support educators and researchers in effectively translating validated educational practices into real-world school contexts.

## All failed implementations are alike

Large-scale projects require sufficient funding and motivated individuals who are committed to making a change, but neither can rescue a project that falls into one or more of the three primary failure modes. A quintessential case study is the technology initiatives of the Milpitas School District and the Los Angeles Unified School District (LAUSD) in the early 2010s. The Milpitas School District utilized strategic technology upgrades (e.g., wireless student devices and corresponding wireless infrastructure) to successfully enhance accessibility, engagement, and learning outcomes for students, while also improving autonomy, professional development, and job satisfaction for teachers. The LAUSD purchased expensive, premium tablet computers for every student and teacher, which imposed significant disruption and provided little benefit^24,25^. While the Milpitas School District had the advantage of being smaller, less complex, and with a better baseline for existing technology infrastructure relative to the LAUSD, and the LAUSD had the advantage of more funding and more connections with industry partners, the resounding success of the Milpitas School District and the infamous failure of the LAUSD replicated the patterns of success and failure observed in other environments.

The superintendent of the Milpitas School District initiated the technology initiative by empowering local teachers and principals, asking, “If you could design the school of the future, what would that look like?”^24,25^. The initiative set clear goals defined by the needs and constraints of students and teachers, focusing on enhancing learning outcomes and achieving equitable access for all students. Careful advanced planning considered the need to support the initiative with corresponding capacity-building efforts, including upgrades to the technology infrastructure and additional professional development training for faculty. A phased roll-out, including small-scale pilot tests of the new technology, revealed the need for adaptations to the original plan. Once the initiative achieved full scale, the district continued to monitor the outcomes and incorporate feedback from teachers and students as part of the regular ongoing operations of the school system. From the early planning stages through incremental testing and to the final assimilation, the project was always collaborative, inquisitive, and iterative. The collaborative aspect was consistent with the principles of Participatory Design^26^ and Learner-Centered Design^27^, which emphasize structured, ongoing, and role-defined involvement of key stakeholders beyond mere consultation. The project supported these collaborative principles through *thorough inquiry* and *iterative revision*, which are essential for successfully adopting these designs during an organizational change process. Thus, the Milpitas School District was able to manage the ill-structured problems it faced.

The LAUSD, in contrast, began its technology initiative not by inquiring about the needs of teachers and students, but by negotiating with high-profile technology vendors. They committed over a billion dollars to purchase expensive devices for every student and then made post-hoc justifications for that choice, with no clear plan for how it would enhance students’ learning^24,25^. These devices were distributed all at once, with no plan for corresponding infrastructure improvements or professional development, and with inadequate monitoring of ongoing developments, let alone adaptation to those developments. In short, they approached the project as though it were a well-structured problem. This led to minimal planning, teamwork, and revision; and thus reproduced the failure modes one would generally expect when making the mistake of approaching an ill-structured problem as though it were well-structured. While both of these California school districts had the same basic idea of upgrading classroom technology, the Milpitas School District achieved improved learning outcomes for students and smoother work processes for teachers, whereas the LAUSD experienced severe disruptions to classroom learning. Although these two California school districts were not identical to each other, the differences in their approach to technology upgrades are highly representative of the best and worst practices for implementing any change in a school context.

These same patterns have been observed in other school contexts where similar changes have been attempted. Whether the change being implemented is a technological upgrade, a pedagogical change, or an attempt at improving equity, similar patterns emerge^6,28–30^. In implementing high-engagement pedagogy in postsecondary education, the most common feature of successful projects is meaningful collaboration between instructors and departmental leadership, especially support for instructors amid early setbacks^31,32^. Other important factors include systemic adaptation within the department to support the change, a formal team to plan and monitor the process, and active communication regarding ongoing setbacks and successes^31,33,34^ Despite the overwhelming evidence that high-engagement pedagogy yields superior learning compared to traditional lecture-based instruction^35^ many efforts to replace lectures with evidence-based instructional practices have been unsuccessful^36^. Though these newer instructional practices unequivocally provide better learning gains, their sustained adoption depends on the process by which they are implemented. These implementation efforts tend to fail when they are haphazardly planned, do not adapt to setbacks, or fail to engage with stakeholders at all levels of the organization.

Drawing on the best and worst practices for crossing the second valley of death from Implementation Science research, the path ahead might seem straightforward: outline clear criteria for discerning between strong and weak plans; describe the known unknowns for anticipating challenges; and provide user-friendly protocols so that researchers, administrators, teachers, and students can function as partners to drive the change. Indeed, the patterns of best and worst practices are so consistent (in education and other contexts)^10,37^, that evidence from Implementation Science provides clear criteria^38,39^ and the open questions in Implementation Science provide the known unknowns^40,41^. The challenging part is consolidating these established findings and open questions in a manner that is both understandable and useful for educators and researchers.

## Consensus in Implementation Science

The findings from Implementation Science are so comprehensive that consolidating them into a single framework would make it too large and complex to serve as a practical guide for planning and conducting an implementation project. Unlike basic science, Implementation Science investigates the intricacy and messiness of real-world human environments. The main challenge is the inherent complexity of itemizing and organizing the numerous underlying factors that lead to the success (or failure) of implementation projects^37^. Since Implementation Science is grounded in diverse theoretical foundations, it has yielded numerous unique frameworks to organize these factors, each with varying emphasis^42^. Some of these frameworks are grounded in behavioral theories that emphasize individual-level factors^43^, while others are grounded in organizational theories that emphasize systemic factors^44^. Amid this diversity of perspectives emerges a remarkable level of agreement that any implementation project must consider both individual-level behavioral factors *and* systemic organizational factors^10,37,38^. Educators and education researchers can therefore use established principles and processes to successfully bridge the second valley of death and implement applied pedagogical solutions into existing educational systems.

Various frameworks emphasize different stages of the implementation process. Since many implementation projects are poorly planned, they often fail before they even begin. Consequently, certain frameworks heavily emphasize processes for creating strong plans at the early stages of the implementation process. And since many other projects trip at the finish line by not adapting the school system (or other organization) to sustain the new intervention in the long term, certain implementation frameworks strongly emphasize the later stages of an implementation project. Again, education reformers have no need to take sides. Although they may emphasize particular points of failure, both sides will agree that points of failure exist in both the early and late stages of the process. Since teachers and students are the best sources of knowledge about their own needs, certain frameworks emphasize a bottom-up structure for teamwork and collaboration. Since most successful projects entail support by leadership and systemic adaptation, some frameworks emphasize a top-down structure for teamwork and collaboration. Yet again, they both agree that all levels of the organization have essential contributions to make.

Many other dimensions of achieving real-world implementation are largely agreed upon across frameworks with varying points of emphasis. Other fields of research have independently confirmed these findings. While the field of Implementation Science has arrived at these findings by studying healthcare systems and educational systems^45,46^, the field of Change Management has arrived at the same findings by studying private corporations^47^. The field of Design Research within cognitive science has arrived at the same findings by studying ill-structured problems in engineering^21,22,48,49^. The field of Project Planning Research (a sub-field of Strategic Management) has also arrived at the same findings^50^. Even narrower fields, such as Physics Education Research, have arrived at the same findings by studying instructional reforms in university physics departments^31^. This consensus in Implementation Science and beyond provides clear criteria for planning an implementation project, but it also presents a challenge in making the findings user-friendly and accessible.

Given this level of agreement, with each framework contributing important components to emphasize, a reasonable approach is to consolidate the various frameworks into a single comprehensive framework^10^. Ultimately, this approach proves to be *too* comprehensive to be practical. One recent consolidated framework uses 39 groups of constructs, encompassing hundreds of individual factors and sub-constructs, to select from 73 implementation strategies^10,41^. A comprehensive list of every possible factor to consider is not easy for anyone to understand, much less can it serve as a practical guide to conducting an implementation project^51^. When given a parsed-out list of this kind, even experts in Implementation Science will not agree on a plan for a given project^40^. Where they agree is that there is a sequence of essential milestones that must be accomplished before moving on to the next^52^. Among those agreed-upon milestones is to meticulously map out the unique advantages and disadvantages present in the context of the project at hand^39^, and then select strategies to leverage the advantages and overcome the disadvantages^37^. This map is what some literature refers to as a Theory of Change (ToC)^53^, and the process for crafting it is a close parallel to SWOT Analysis (Strengths, Weaknesses, Opportunities, and Threats) from Strategic Management literature^54,55^. The ToC discussed here is different from those typically required in education research grants, which are a simpler mapping of mechanisms of change and outcomes with little specification of much else; they rarely identify the potential disadvantages/ challenges or need for adaptation. Where there is open disagreement and ongoing research within Implementation Science is how to select specific strategies that address the unique contextual factors of any given project. Herein lies the set of known unknowns that the education research community needs to consider. Going into any implementation project, it is possible to anticipate some, but not all, components of the plan. When implementing a new teaching methodology, one can anticipate the need to train instructors on its use, and that this extra workload for the instructors will strain their work capacity. However, it is impossible to anticipate the exact best way to help them and the school as a whole adapt to this strain. Where it is not possible to anticipate the best strategy, the plan must anticipate challenges and the need for ongoing revision to the plan.

While some experts in the field of Implementation Science possess the training and experience to utilize consolidated frameworks with lengthy, detailed lists of every factor to consider and every possible strategy in response to those factors, most education researchers, educators, and school administrators certainly do not. We argue that teachers and students can never provide genuine involvement in the process if systemic change efforts are planned and conducted using frameworks that were not designed for them to understand. Thus, if we “talk the talk” of stakeholder engagement and empowerment, we must “walk the walk” by providing user-friendly protocols.

## A practical protocol to guide implementations

To make our protocol user-friendly, we have designed it to avoid jargon, outline a step-by-step process, and include both concrete guidelines and broad principles for overcoming the most common failure modes. We also integrate and simplify existing frameworks and planning tools rather than creating yet another new framework. These simplified tools are provided in the supplementary information. Therein, we provide readers with a one-page summary of the principles, processes, and pitfalls, which serves as an introductory overview of a typical implementation process. Along with this, we provide a checklist for planning and conducting an implementation project that is patterned after familiar pre-registration protocols. This checklist provides criteria for advancing from one stage of the process to the next. Also, we provide a brainstorming aid to help craft a Theory of Change for an implementation project. This brainstorming aid is based on SWOT Analysis^54,55^. Finally, we provide brief accounts of three well-documented case studies illustrating key factors leading to success and failure in implementation projects. Since planning and conducting an implementation project is an ill-structured problem, these tools in the supplementary information do not include a list of every factor to consider. Rather, they provide a basis to ensure that the planning is sufficiently thorough in *finding* factors to consider.

**Fig. 2:**
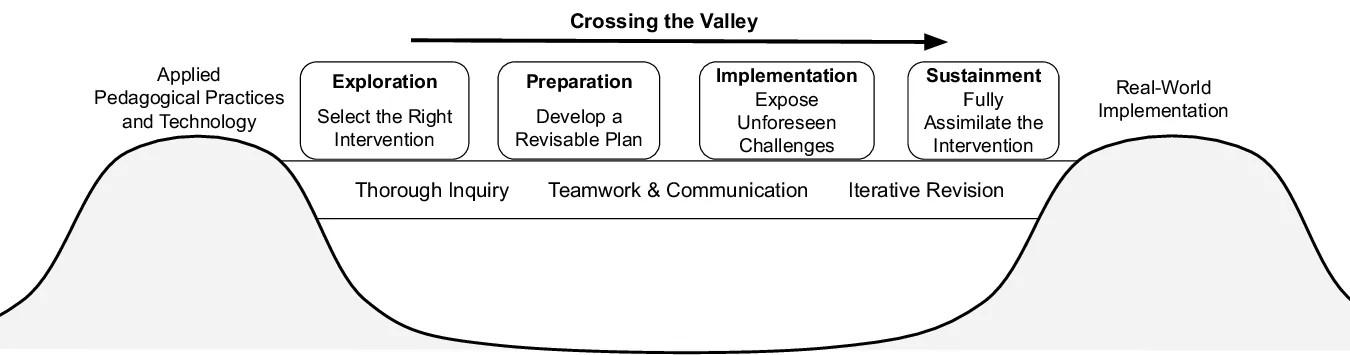
The broad principles and the concrete milestones are all essential for successfully implementing research-validated pedagogical practices and technologies in schools. Without thoughtfully adhering to the principles, it is possible to check off each milestone in a disingenuous manner. Without meticulously achieving the concrete milestones, it is possible to have the proper mindset for success while inadvertently setting up the project for failure. Each milestone must be achieved before moving on to the next, and each must be supported by all three principles to be achieved to a sufficiently high standard.

Since any implementation project must cultivate collaboration before it can collaboratively make a plan, and it must make a plan before it can adapt to unforeseen challenges in enacting that plan, it might seem that the three major failure modes would arise in this sequence: first, lack of stakeholder engagement; then, poor planning; and finally, lack of adaptation. Counterintuitively, all three of these failure mechanisms can arise at key points throughout the process^6,11,56^. Thus, the approach we propose is to break down the process into a series of four essential milestones; and to rigorously ensure that each milestone has been fully met and that the major failure modes have been avoided before moving on to the next milestone (the checklist in the supplementary information outlines key milestones and how to evaluate progress towards them). These four milestones correspond to four distinct phases, which provide a sequence of building blocks to cross the valley between applied science and successful implementations (Fig. 2):

- **Exploration Phase:** Selecting the right intervention requires forming partnerships with students and teachers, and understanding their needs first.
- **Preparation Phase:** Developing a revisable plan requires collaboratively mapping out a Theory of Change (ToC) to help predict how the new intervention should work in context and the complexities that could arise throughout the process.
- **Implementation Phase:** Exposing unforeseen challenges requires small-scale testing and support for teachers amid early setbacks.
- **Sustainment Phase:** Assimilating the new intervention into the regular operations of the educational system requires subjecting it to the existing quality assurance mechanisms in use.

There are various ways to delineate between the end of one phase and the beginning of another. We have chosen to depict it using the EPIS Framework (Exploration, Preparation, Implementation, and Sustainment) because it has two planning phases, one for exploration and another for preparation, whereas other process-based frameworks have only one planning phase^38^. The two planning phases encourage implementation projects to pursue a high degree of preparation, and they encourage funding proposals to specify activities for in-depth planning and preparation. A successful implementation typically requires a level of time-consuming, resource-intensive planning that exceeds what can be expected in advance of submitting most grant proposals^38^. Any project becomes easier to navigate when guided by both concrete milestones and broad principles. To this end, we offer a positive reframing of the three main failure modes in the form of three complementary principles for success (Fig. 2):

- **Thorough Inquiry:** Successful projects continually strive to gain a deep understanding of students’ needs, explore multiple possible ways to meet those needs, consider the unique constraints in the local context, and closely monitor for unforeseen issues.
- **Teamwork & Communication:** Successful projects have clear team member roles, make all important decisions in partnership with students and teachers, and openly share setbacks and successes.
- **Iterative Revision:** Successful projects iterate within each phase until meeting the milestones to a high standard, set flexible timelines and budgets, and adapt their plan in response to new information.

All three principles for success require an intentional commitment of time and resources. Successful projects allocate a budget to actively build a team with clear lines of communication, and formal roles for stakeholders on the team, along with formal roles for the co-investigators, project staff, and leadership on the team. Successful projects also allocate time and resources to understand the problem before committing to a solution, and to iteratively revise the solution. Not surprisingly, the three failure modes are more likely to occur in cases where time and resources are not specifically allocated in those areas. We urge educators and researchers to recognize implementation projects as ill-structured problems and to allocate what time and resources they have into the necessary inquiry, collaboration, and revision.

## From evidence to impact: lessons, limitations, and next steps

The challenge of translating educational research findings into practical applications is a persistent and significant barrier to achieving large-scale educational innovation. While many educators and researchers recognize this issue, a substantial disconnect remains between understanding what needs to be done and successfully implementing it in real-world settings. This gap often arises not from a lack of evidence-based solutions but from inadequate understanding and execution of the implementation process itself. This perspective is especially crucial for researchers and educators who may be less familiar with Implementation Science methodologies but are eager to see their findings and innovations succeed in real-world educational contexts.

The practical framework outlined in this paper aims to simplify the complexities inherent in implementing educational research findings. Our goal is not to suggest that implementing research findings in educational settings is straightforward—indeed, it remains a challenging endeavor—but rather to provide clear, user-friendly guidance that leverages established knowledge from implementation science in a straightforward and approachable way. By distilling a vast body of research into accessible tools (provided in the supplementary information), we hope to equip education professionals with actionable strategies to anticipate, plan for, and navigate the known challenges inherent in educational reform and implementation efforts.

However, even with simplified protocols, successfully bridging the gap between applied research and practical implementation remains a significant undertaking. Our protocol emphasizes critical success factors, including detailed planning, proactive adaptation, and robust teamwork and communication, each of which requires dedicated time, resources, and continuous attention. Simplification does not eliminate complexity; rather, it aims to reduce the barriers to entry, making rigorous planning and adaptation feasible for a wider range of researchers and educators. Therefore, we encourage readers to approach implementation efforts with humility, openness to collaboration, and readiness for iterative revision.

To get started, education researchers and practitioners should first cultivate authentic partnerships with local stakeholders—including students, teachers, administrators, and families—to clearly understand and articulate local needs. Next, they should collaboratively explore multiple intervention options and rigorously assess their compatibility with the unique cultural, structural, and systemic characteristics of their specific context. Planning should not only identify initial steps but also explicitly budget time, resources, and personnel to continuously revise and adapt strategies based on ongoing feedback.

It is also crucial to recognize that simplified protocols, although useful, have limitations. The simplification necessary to increase accessibility also omits the depth and nuance present in more comprehensive frameworks. Hence, practitioners should view the protocols provided here as a solid foundation, rather than a definitive or exhaustive manual. Those who wish to deepen their understanding or address more complex issues are encouraged to engage further with the broader Implementation Science literature or consult specialists in the field.

Ultimately, we acknowledge that the transition from promising research application to successful implementation is inherently complex and challenging. Yet, despite these challenges, we remain optimistic. The clear consistency in Implementation Science across diverse fields provides both a warning and an opportunity: a warning against simplistic assumptions about real-world change, and an opportunity to build systematically on what is already well understood. With appropriate planning, attention to context, meaningful stakeholder engagement, and structured yet flexible protocols, education professionals have a robust starting point from which to effect lasting, meaningful change.

In conclusion, we urge funding agencies and education policy-makers to align their standards and resources accordingly, incentivizing and rewarding rigorous implementation planning and recognizing the genuine complexity involved in translating research into impactful educational practice. By doing so, they will play a crucial role in bridging the gap between educational research and practice, ultimately enhancing the positive impact on teaching, learning, and student well-being globally.

## Supplementary information

Supplementary information

## Data Availability

No datasets were generated or analyzed during the current study.
