# Supplementary material for: A practical implementation science framework for education
Source: NPJ Sci Learn. 2026 Jun 18;11:51. doi: 10.1038/s41539-026-00430-w (PMC13638982; doi:10.1038/s41539-026-00430-w)
Supplement: Supplementary file 1 [file 41539_2026_430_MOESM1_ESM.pdf]

Supplementary Note 1:
One-Page Cheat Sheet for Successful School Reforms

Successful reforms can have many different starting points, but they tend to achieve success in a common fashion. Some reforms start in times of crisis, while others capitalize on opportunities. Some are initiated by leadership, while others have grassroots origins. From any of these starting points, successful reforms quickly involve contributions at all levels of the organization, especially meaningful support by leadership for teachers. Time and resources must be applied to team building and communication. While it is common to adapt the intervention to match the context, most successful reforms also make systemic adaptations to the school in support of the new intervention. Expect setbacks along the way. Reforms that thrive proactively adapt to these setbacks. Strong planning enables adaptation. The principles for success are familiar principles for solving any ill-structured problem.

Three Core Principles

1. Thorough Inquiry
Brainstorm multiple possible solutions
Study similar reforms and interventions
Test the intervention to expose challenges

2. Teamwork & Communication
Involve all levels of the organization
Communicate early successes and setbacks
Provide real support when challenges arise

3. Iterative Revisions
Learn from unexpected outcomes
Use flexible timelines and budgets
Make a detailed Theory of Change

As with all ill-structured problems, successful reforms begin with a thorough understanding of the problem, then shift to iterative testing and revision of possible solutions. It culminates in assimilating the solution into the school's ordinary operations. The reform can advance more quickly and with more success by breaking it down into focused phases. Each phase lays the groundwork for the next by stepping through specific processes that build essential components of the team, the theory, and the solution along the way.

Products

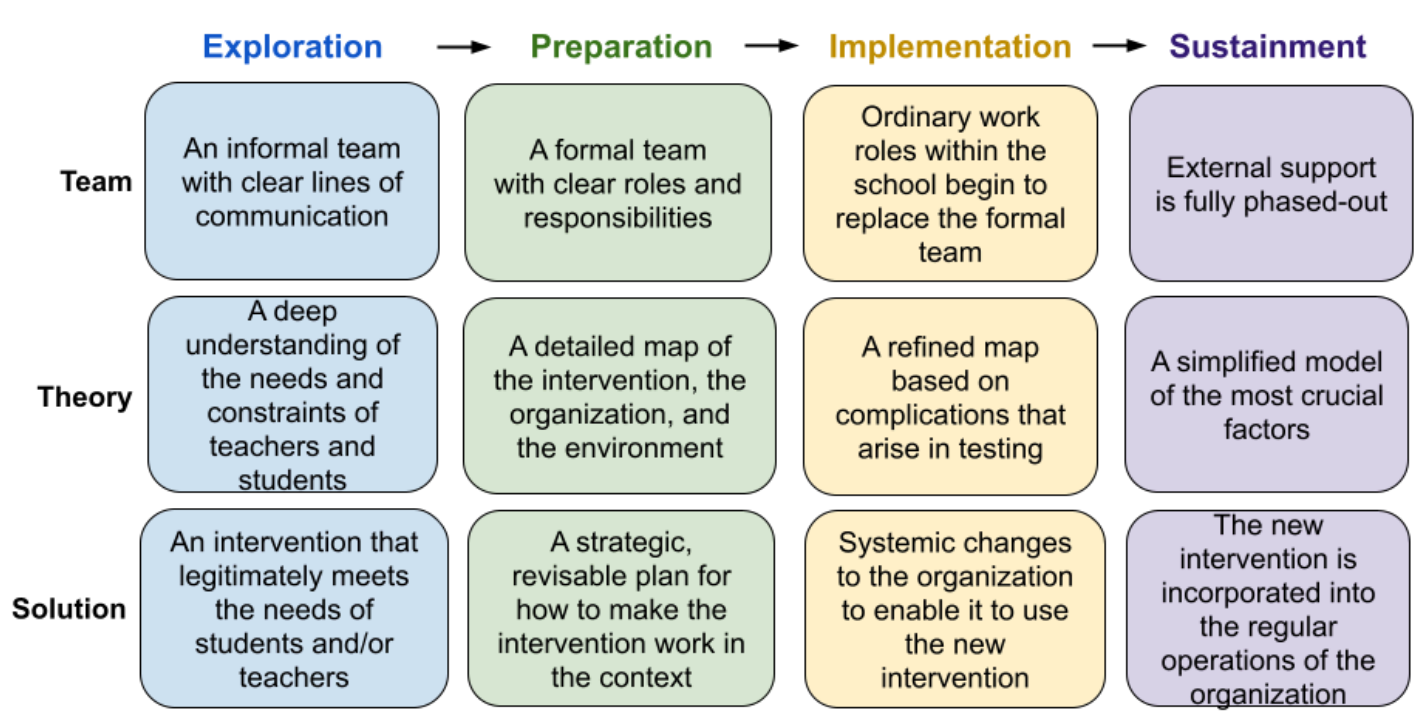

Processes

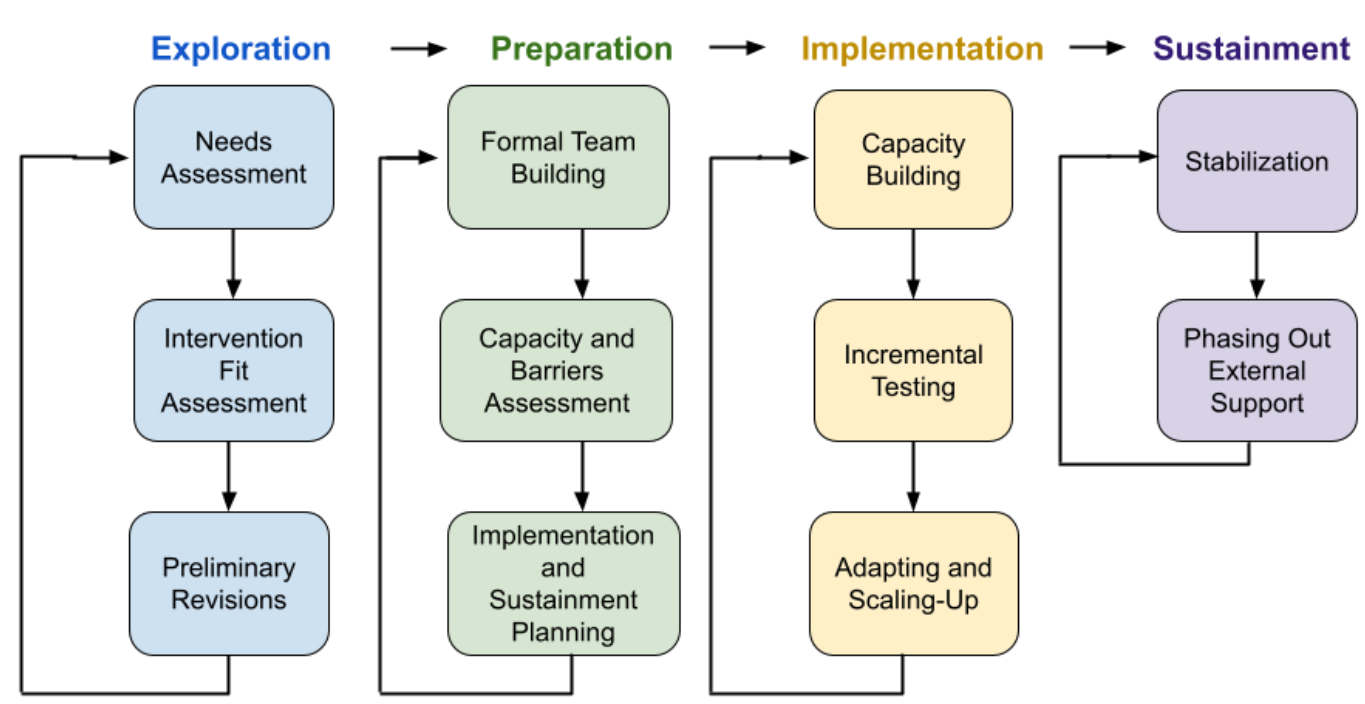

Needs Assessment: Brainstorm and explore a wide range of local needs before selecting an intervention
Intervention Fit Assessment: Propose multiple possible solutions and scrutinize compatibility with the unique context
Preliminary Revisions: Explore several possible adaptations of the intervention to meet the needs and fit the context
Formal Team Building: Outline clear roles and lines of communication among local school members and external experts
Capacity and Barriers Assessment: Plan for addressing issues related to budgets, infrastructure, workload, expertise, etc.
Implementation and Sustainment Planning: Craft plans to build capacity and adapt to setbacks throughout the process
Capacity Building: Update budgets, infrastructure, workload, training, etc., as needed to make use of the new intervention
Incremental Testing: Conduct small-scale tests and observe results to scale up progressively
Adapting and Scaling Up: Incorporate lessons learned into adapting the team, the theory, and the solution
Stabilization: Incorporate the intervention into the regular operations of the school organization
Phasing Out External Support: Gradually allow the local organization to take full responsibility for using the intervention

Though each reform faces unique challenges, reforms that fail often fall into a common set of foreseeable pitfalls. These pitfalls can arise when approaching any ill-structured problem as though it is well-structured.

Three Common Pitfalls

1. Simplistic Planning
Post-hoc justification of a pre-selected solution
Exceeding organizational capacity

2. Poor Teamwork
Unclear roles within the team
Failure to utilize local knowledge

3. Lack of Adaptation
Failure to monitor progress
Insufficient resources for testing and revision

For in-depth guidance, see Allen, S., Bardach, L., Jirout, J., Mackey, A., McCoy, D., Pesando, L. M., & Kizilcec, R. (2025). Implementing new technology in educational systems. In T. S. Behrend (Ed.), Human-technology partnerships at work (pp. 259–283). Cambridge University Press. Reproduced with permission of The Licensor through PLSclear.

Supplementary Note 2:
Milestones and Readiness Indicators for School Reforms

Major milestones in each of the four EPIS phases, and indicators of readiness to move to the next phase. Though the overall process is mostly linear, with each phase producing essential preparation for the next, each individual phase is cyclical. The sequence of steps within each phase should be iterated upon until meeting the milestone of that phase to a high standard. The readiness indicators don't indicate if the idea behind the new intervention is correct. Rather, they indicate whether processes and principles have been used with a sufficient degree of rigor.

**Exploration Phase**	**Preparation Phase**	**Implementation Phase**	**Sustainment Phase**
Major Milestones			
**Selected an intervention that meets local needs**<br>○ Collaborated with local stakeholders to outline local needs<br>○ Brainstormed multiple possible interventions to meet those needs<br>○ Made preliminary revision to the selected intervention to fit the context	**Developed a revisable strategic plan**<br>○ Built a team representing all levels of the school organization<br>○ Mapped out the advantages and disadvantages within the organization (i.e., a Theory of Change [ToC])<br>○ Planned for the implementation and sustainment phases	**Exposed unforeseen challenges and iteratively refined the plan**<br>○ Built the capacity of the organization to use the intervention<br>○ Conducted small-scale tests<br>○ Openly communicated successes and setbacks<br>○ Adapted the plan, revised the intervention, and scaled-up tests	**Incorporated the new intervention into the regular operations of the organization**<br>○ Imposed stability mechanisms and quality assurance processes<br>○ Phased out external support
Readiness Indicators			
○ New challenges and opportunities were identified during the needs assessment<br>○ Local stakeholders made specific contributions to the needs assessment<br>○ Conflicting needs were identified during the needs assessment<br>○ Local stakeholders made specific contributions to the intervention fit assessment	○ Local stakeholders from all levels of the organization have clear roles in the project and have made specific contributions<br>○ School leadership is ready to offer support when setbacks occur<br>○ ToC includes factors from the intervention, the organization, and the environment in which the organization operates<br>○ ToC includes the causal mechanisms that make the intervention work<br>○ ToC includes stability mechanisms and quality assurance processes<br>○ The plan allocates time and resources for testing and revision	○ Unforeseen complications exposed via small-scale testing led to specific revisions to the intervention, to capacity-building strategies, and the ToC<br>○ Local stakeholders made specific contributions to these revisions	○ The new intervention does not strain the capacity of the organization in terms of budgets, workloads, training, or infrastructure<br>○ Local stakeholders at all levels of the organization have a shared understanding of their role in using and maintaining the new intervention for the long term.

For in-depth guidance, see Allen, S., Bardach, L., Jirout, J., Mackey, A., McCoy, D., Pesando, L. M., & Kizilcec, R. (2025). Implementing new technology in educational systems. In T. S. Behrend (Ed.), Human-technology partnerships at work (pp. 259–283). Cambridge University Press. Reproduced with permission of The Licensor through PLSclear.

Supplementary Note 3:
SWOT Analysis for School Reforms

School reforms require a Theory of Change to inform planning, testing, and revision. A good ToC accounts for the interactions of the new intervention with the unique context of the school organization and the broader environment. Since a school reform is an ill-structured problem, there is no replacement for effortful forethought about how to make the solution work in the unique context. Brainstorming the unique advantages, disadvantages, and corresponding strategies is a great opportunity to build partnerships with teachers and students.

1. Brainstorm factors to fill in the SWOT table in collaboration with all levels of the school organization.
2. Carefully outline interactions among these factors and prioritize them based on the potential impact on the project.
3. Develop strategies to overcome barriers and leverage advantages. Anticipate revision.

	**Strengths & Opportunities**	**Weaknesses & Threats**
**Intervention**<br><br>Scalability<br>Adaptability<br>Context Dependence<br>Cost<br>Technology Constraints<br>Required Expertise<br>Causal Mechanisms<br>Etc.		
**Organization**<br><br>Leadership Structure<br>Staffing<br>Training and Expertise<br>Emotional Factors<br>Policy and Procedures<br>Technological Capacity<br>Budgets<br>Etc.		
**External Environment**<br><br>Cultural<br>Political<br>Socioeconomic<br>Legal<br>Etc.		

For in-depth guidance, see Allen, S., Bardach, L., Jirout, J., Mackey, A., McCoy, D., Pesando, L. M., & Kizilcec, R. (2025). Implementing new technology in educational systems. In T. S. Behrend (Ed.), Human-technology partnerships at work (pp. 259–283). Cambridge University Press. Reproduced with permission of The Licensor through PLSclear.

Supplementary Note 4: Case Studies of School Reforms

Case 1: Active Learning in University Physics Courses

Efforts to replace traditional lecture-based instruction with interactive engagement pedagogy in university physics courses have yielded remarkable improvements in learning gains, reduced achievement gaps, and boosted student satisfaction (Hake, 1998; Beichner et al., 2007; Freeman et al., 2014; Theobald et al., 2020; Lorenzo et al., 2006). Despite the strength and maturity of this evidence base, successful and sustained implementation has proven uneven across institutions

One of the most extensively documented successes is the Student-Centered Active Learning Environment with Upside-down Pedagogies (SCALE-UP) project originating at North Carolina State University (Beichner, 2000; Beichner et al., 2007). A defining feature of this effort was early and sustained adaptation at the departmental level. Physical classroom infrastructure was redesigned to support peer collaboration, and departmental leadership provided explicit, visible support to instructors during early implementation, including periods of student dissatisfaction and initially flat assessment outcomes. Faculty involved in the reform were protected from premature negative evaluation, particularly in tenure and promotion contexts, and were granted reduced teaching loads to allow time for course redesign and professional development (Henderson & Dancy, 2007; Foote et al., 2016; Wieman, 2017). Collective ownership of course material was codified in departmental policy, ensuring continuity when courses transitioned to new instructors and reducing the need to relearn hard-won implementation lessons (Pollock & Finkelstein, 2008; Henderson et al., 2012).

Across less successful implementations, similar reforms failed to survive early setbacks or gradually reverted to lecture-based instruction over time (Henderson & Dancy, 2007; Henderson et al., 2012; Pollock & Finkelstein, 2008). These implementation efforts were often led by individual instructors in isolated courses, with limited or no formal involvement from department leadership (Henderson et al., 2011). A recurring issue was that standard student feedback instruments are particularly sensitive to the predictable frustration associated with implementation trials, creating perceived evaluative risk for pre-tenure faculty (Wieman, 2017). A second, related issue arises when reformed courses transition to new instructors-of-record without any formal expectation to retain the high-engagement materials or pedagogical practices developed by their predecessors. Both dynamics are substantially exacerbated in the absence of clearly assigned departmental responsibility for overseeing and sustaining instructional reforms (Foote et al., 2016; Henderson et al., 2011).

Effective implementation of active learning in university physics requires enacting reforms at the departmental level and making key systemic adaptations in advance of implementation. In particular, the role of departmental leadership in endorsing, protecting, and sustaining the reform should be formally codified before the project begins. Providing incentives rather than risks to pre-tenure faculty is essential. Finally, student feedback instruments should be revised to focus on aspects of instruction that students are well positioned to observe, rather than asking them to evaluate their own learning, thereby reducing predictable distortions in evaluative signals during early implementation.

Supplementary Note 4: Case Studies of School Reforms

Case 2: Restorative Discipline in K-12 Education

Projects that augment exclusionary discipline practices (e.g., suspension and expulsion) with restorative approaches (e.g., mentoring, counseling, and facilitated dialogue) have documented reductions in serious disciplinary incidents, improvements in student–teacher trust and classroom climate, and in some contexts reductions in racial disparities in disciplinary action (Gregory & Evans, 2020; Jain et al., 2014; Fronius et al., 2016; Fronius et al., 2019; Okilwa, 2022). Importantly, these benefits depend less on the formal policy change itself than on the quality of implementation.

A well-documented success is the Oakland Unified School District (Jain et al., 2014; Fronius et al., 2019), which invested in sustained, two-way communication with teachers and parents and structured implementation around phased pilots, outcome monitoring, and planned adaptation. Early trials revealed that restorative practices were substantially more time-intensive and emotionally demanding than anticipated, and that initial guidance left ambiguity regarding teachers' authority and classroom management responsibilities (Gregory & Evans, 2020). Teachers reported an unintended burden to resolve interpersonal conflicts beyond classroom-level control, while parents sometimes interpreted restorative practices as a reduction in accountability (Gregory & Evans, 2020). Because these predictable challenges were surfaced early and addressed collaboratively, the district made targeted revisions: restorative practices were positioned as a supplement rather than a replacement for standard discipline, additional staffing was allocated to manage workload, and teachers' disciplinary roles were clarified (Jain et al., 2014).

Across less successful implementations, restorative discipline policies were often rolled out at full scale with minimal pilot testing and weak feedback channels for collaborating with teachers on practical constraints (Fronius et al., 2019; Gregory & Evans, 2020; ASTHO). In some cases, pilots functioned primarily as proof-of-concept demonstrations rather than as diagnostic phases designed to expose limitations and trigger adaptation, and therefore lacked systematic outcome monitoring. These efforts frequently treated restorative practices as a near-complete substitute for conventional discipline. While motivated by similar equity-oriented goals, failure to revise the approach in response to early challenges led to increased behavioral issues, deterioration of classroom climate, and erosion of teacher trust and buy-in (Fronius et al., 2019).

Effective implementation requires using early trials as a genuine diagnostic phase, with explicit plans for monitoring outcomes and collaborating with educators and families to revise policy. Because many of the practical constraints in restorative discipline reforms are relational rather than technical, formal planning and monitoring protocols must be paired with sustained relational work (i.e., clarifying roles, supporting teachers' authority, and communicating clearly with parents) to prevent predictable tensions from undermining implementation and long-term sustainment (Bartley, 2022; Gregory, 2016).

Supplementary Note 4: Case Studies of School Reforms

Case 3: Collaborative Learning in K-12 Education

Projects to replace teacher-centered instruction with structured collaborative learning approaches in K-12 classrooms have documented improvements in academic achievement, student engagement, and peer relations, with more equitable participation observed in the most carefully implemented cases (Slavin, 1995; Slavin, 1996; Roseth, Johnson, & Johnson, 2008; Cohen & Lotan, 1997). As with other instructional reforms, the magnitude and durability of these benefits depend critically on implementation quality rather than on adoption of the instructional model alone.

Across successful implementations,collaborative learning was introduced at the school or district level, with explicit leadership support for teachers and shared ownership of instructional practices and materials (Johnson, Johnson, & Holubec, 1994; Slavin, 1995; Durlak & DuPre, 2008). Professional development was treated as ongoing instructional infrastructure rather than as a one-time orientation, and implementation proceeded gradually, allowing challenges to surface and be addressed through planned adaptation (Johnson et al., 1994; Slavin, 1995; Cohen & Lotan, 2014). One key challenge that arose was implementation drift: in response to the practical burdens of collaborative instruction, classrooms tended to shift toward less structured group activities, thereby weakening or eliminating the causal mechanisms such as individual accountability and structured interdependence (Slavin, 1995; Slavin,1996). In successful cases, this drift was countered through targeted guidance on appropriate use, including clarification of which topics were most suitable for collaborative methods and how structure should be maintained in practice (Johnson, Johnson, & Holubec, 1994; Slavin, 1995). These refinements were incorporated into routine school operations, enabling sustained, high-fidelity use of the instructional approach (Durlak & DuPre, 2008).

In less successful implementations, drift toward unstructured group work was identified but not adequately supported. Teachers were often instructed to correct implementation problems without additional time, training, or curricular support, leading to further erosion of essential design features (Slavin, 1995; Slavin, 1996). In some of the most severe cases, this loss of structure occurred amid explicit advocacy for minimal instructional guidance, in which reduced structure was not treated as an implementation failure but as a deliberate design choice grounded in broad theoretical commitments about learning (Kirschner, Sweller, & Clark, 2006). Whether due to superficial understanding of collaborative learning methods or deliberate theoretical alignment, these approaches contradicted extensive empirical evidence that effective collaborative learning requires substantial and sustained instructional scaffolding (Slavin, 1996).

Effective implementation of collaborative learning requires explicit identification and protection of the causal mechanisms by which the reform produces learning gains. Planning must anticipate implementation drift and allocate resources for ongoing professional development, monitoring, and targeted adaptation. Successful reforms attend not only to the adoption of collaborative activities, but to the systemic supports necessary for teachers to include and maintain essential causal mechanisms.

References

Beichner, R. J. (2000). The Student-Centered Active Learning Environment for Undergraduate Programs (SCALE-UP). North Carolina State University.

Beichner, R. J., Saul, J. M., Abbott, D. S., Morse, J. J., Deardorff, D., Allain, R. J., Bonham, S. W., Dancy, M. H., & Risley, J. S. (2007). The Student-Centered Activities for Large Enrollment Undergraduate Programs (SCALE-UP) project. Research-Based Reform of University Physics, 1(1), 2–39.

Bartley, T. (2022). Rules without rights: Land, labor, and private authority in the global economy. Oxford University Press.

Cohen, E. G., & Lotan, R. A. (1997). Working for equity in heterogeneous classrooms: Sociological theory in practice. Teachers College Press.

Cohen, E. G., & Lotan, R. A. (2014). Designing groupwork: Strategies for the heterogeneous classroom (3rd ed.). Teachers College Press.

Durlak, J. A., & DuPre, E. P. (2008). Implementation matters: A review of research on the influence of implementation on program outcomes. American Journal of Community Psychology, 41(3–4), 327–350.

Foote, K. T., Knaub, A. V., Henderson, C., Dancy, M. H., & Beichner, R. J. (2016). Enabling and challenging factors in institutional reform: The case of SCALE-UP. Physical Review Physics Education Research, 12(1), 010103.

Freeman, S., Eddy, S. L., McDonough, M., Smith, M. K., Okoroafor, N., Jordt, H., & Wenderoth, M. P. (2014). Active learning increases student performance in science, engineering, and mathematics. Proceedings of the National Academy of Sciences, 111(23), 8410–8415.

Fronius, T., Persson, H., Guckenburg, S., Hurley, N., & Petrosino, A. (2016). Restorative justice in U.S. schools: A research review. WestEd.

Fronius, T., Persson, H., Guckenburg, S., Hurley, N., & Petrosino, A. (2019). Restorative justice in education: What we know so far. WestEd.

Gregory, A. (2016). The promise of restorative practices to transform teacher–student relationships and achieve equity in school discipline. Journal of Educational and Psychological Consultation, 26(4), 325–353.

Gregory, A., & Evans, K. R. (2020). The starts and stumbles of restorative justice in education: Where do we go from here? National Education Policy Center.

Supplementary Note 4: Case Studies of School Reforms

Hake, R. R. (1998). Interactive-engagement versus traditional methods: A six-thousand-student survey of mechanics test data. American Journal of Physics, 66(1), 64–74.

Henderson, C., & Dancy, M. H. (2007). Barriers to the use of research-based instructional strategies: The influence of both individual and situational characteristics. Physical Review Special Topics – Physics Education Research, 3(2), 020102.

Henderson, C., Beach, A., & Finkelstein, N. (2011). Facilitating change in undergraduate STEM instructional practices: An analytic review of the literature. Journal of Research in Science Teaching, 48(8), 952–984.

Henderson, C., Finkelstein, N., & Beach, A. (2012). Beyond dissemination in college science teaching: An introduction to four core change strategies. Journal of College Science Teaching, 42(1), 18–25.

Jain, S., Bassey, H., Brown, M. A., & Kalra, P. (2014). Restorative justice in Oakland schools: Implementation and impacts. Oakland Unified School District.

Johnson, D. W., Johnson, R. T., & Holubec, E. J. (1994). The new circles of learning: Cooperation in the classroom. Association for Supervision and Curriculum Development.

Kirschner, P. A., Sweller, J., & Clark, R. E. (2006). Why minimal guidance during instruction does not work. Educational Psychologist, 41(2), 75–86.

Lorenzo, M., Crouch, C. H., & Mazur, E. (2006). Reducing the gender gap in the physics classroom. American Journal of Physics, 74(2), 118–122.

Pollock, S. J., & Finkelstein, N. D. (2008). Sustaining educational reforms in introductory physics. Physical Review Special Topics – Physics Education Research, 4(1), 010110.

Roseth, C. J., Johnson, D. W., & Johnson, R. T. (2008). Promoting early adolescents' achievement and peer relationships: the effects of cooperative, competitive, and individualistic goal structures. Psychological bulletin, 134(2), 223.

Slavin, R. E. (1995). Cooperative learning: Theory, research, and practice (2nd ed.). Allyn & Bacon.

Slavin, R. E. (1996). Research on cooperative learning and achievement: What we know, what we need to know. *Contemporary Educational Psychology*, 21(1), 43–69.

Theobald, E. J., Hill, M. J., Tran, E., Agrawal, S., Arroyo, E. N., Behling, S., … Freeman, S. (2020). Active learning narrows achievement gaps for underrepresented students. Proceedings of the National Academy of Sciences, 117(12), 6476–6483.

Supplementary Note 4: Case Studies of School Reforms

Wieman, C. (2017). Improving how universities teach science: Lessons from the science education initiative. Harvard University Press.
